# HiLight-PTM: an online application to aid matching peptide pairs with isotopically labelled PTMs

**DOI:** 10.1093/bioinformatics/btz654

**Published:** 2019-08-19

**Authors:** Harry J Whitwell, Peter DiMaggio

**Affiliations:** Department of Chemical Engineering, Imperial College London, London, SW7 2AZ, UK; Department of Chemical Engineering, Imperial College London, London, SW7 2AZ, UK

## Abstract

**Motivation:**

Database searching of isotopically labelled PTMs can be problematic and we frequently find that only one, or neither in a heavy/light pair are assigned. In such cases, having a pair of MS/MS spectra that differ due to an isotopic label can assist in identifying the relevant *m*/*z* values that support the correct peptide annotation or can be used for *de novo* sequencing.

**Results:**

We have developed an online application that identifies matching peaks and peaks differing by the appropriate mass shift (difference between heavy and light PTM) between two MS/MS spectra. Furthermore, the application predicts, from the exact-match peaks, the mass of their complementary ions and highlights these as high confidence matches between the two spectra. The result is a tool to visually compare two spectra, and downloadable peaks lists that can be used to support *de novo* sequencing.

**Availability and implementation:**

HiLight-PTM is released using shinyapps.io by RStudio, and can be accessed from any internet browser at https://harrywhitwell.shinyapps.io/hilight-ptm/.

**Supplementary information:**

Supplementary data are available at *Bioinformatics* online.

## 1 Introduction

The use of stable isotopes to heavy label proteins (e.g. SILAC) is a common proteomic approach (Chen *et al.*, 2015), allowing the simultaneous analysis of multiple samples whilst reducing experimental variation. In such an experiment, proteins may be analyzed by tandem mass spectrometry (MS/MS) and peptide fragmentation spectra searched against a database of *in silico* fragmentation peak lists for proteins of interest. It is common that many of the peptides’ fragmentation spectra will go unassigned, for example due to mixed spectra arising from co-fragmentation, fragment ions having too low an intensity, isotopically labelled amino acids having ambiguous masses or the presence of post-translational modifications (Hart-Smith *et al.*, 2016). In such cases, having a pair of MS/MS spectra, that differ due to an isotopic label or post translational modification, can assist in identifying the relevant *m/z* values that support the correct peptide annotation.

**Fig. 1.**
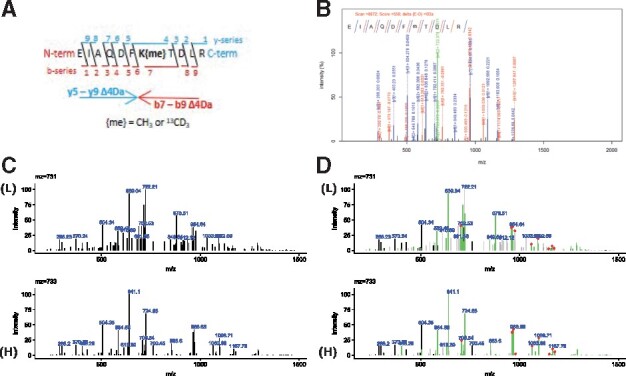
Example application for comparing MS/MS fragmentation spectra for a heavy/light methylated peptide pair. (**A**) N-terminal (b-ions) and C-terminal (y-ions) series for a histone H3 peptide containing a methylated lysine. The fragments that will contain the methylated lysine will have a difference of Δ4Da (the difference between CH_3_ and ^13^CD_3_). (**B**) Database searching identified the heavy-labelled peptide, but not the light. The sequence is provided at the top of the panel; m = monomethyl-lysine and N-terminus is propionylated. (**C**) Original fragmentation spectra for the light (L) and heavy (H) methylated peptide. (**D**) Comparison of the two fragmentation spectra showing exact-matched ions (black), peaks differing by the expected mass shift (green) and those peaks that have a mass shift and have been predicted from the exact-matched ions (red points)

Consider the histone H3 peptide ‘EIAQDFK_{me}_TDLR’ where the lysine is monomethylated and MS/MS spectra exist for both the light (CH_3_) and heavy (^13^CD_3_) methylated peptide. The y and b ion series for both spectra will match prior to the methylated lysine (i.e. y-ions 1-4 and b-ions 1-6). After the methyl lysine, the fragmentation series will differ by 4 Da (i.e the difference between heavy and light methylation) (Fig. 1A). In studies using heavy labelled PTMs [e.g. methylation (Ong *et al.*, 2004; Zee *et al*, 2010), acetylation (Evertts *et al.*, 2013)], the difference in mass between the heavy and light modification is known in advance. The *m/z’*s that match exactly between the two spectra can be used to predict what *m/z’*s should be present if that modification was there, and the supporting y and b ion can be identified.

We have developed an online web application to compare two MS/MS spectra. The application graphically presents the spectra showing *m/z’*s that match exactly or are separated by the specified mass shift, with added confidence if they are calculated from their complementary ion series.

The user is able to adjust the graph in order to output high-quality figures as well as download the matched peak lists that may be used for *de novo* sequencing.

## 2 Description

The application, available online at https://harrywhitwell.shinyapps.io/hilight-ptm/, was created in R (version 3.5.0) as a shiny (1.1.0) app that uses packages ggplot2 (3.0.0) and gridExtra (2.3) to generate and output plots. It takes one or two comma separated value (csv) files as input, each containing a peak list from a centroided MS/MS scan with *m/z* and intensity values in the first and second columns respectively.

The peak list is filtered by binning the *m/z* values and returning the most intense values in each bin (Cox *et al.*, 2011). The size of the bin (parameter: Bin width) and the number of peaks in each bin (parameter: Number per bin) set as 100 and 10 respectively by default.

The user is able to display the MS/MS for the first peak list provided, the second or both with their x-axis aligned. Peak lists are tabulated underneath.

If two peak lists are provided, the user can toggle peak matching which will identify *m/z* values that are shared between the two spectra within a customizable tolerance (0.4 *m/z* by default). Matched peaks are displayed on the MS/MS plot as coloured lines, and the unmatched peaks can be gradually removed using the line-fade slider under plot aesthetic options.

The user may also toggle identification of *m/z* values that are mass-shifted (i.e. the ion series that incorporates the modified amino acid), by selecting ‘Match Mass-shift’ and entering the isotopic difference between the heavy and light PTM (Δ) and the precursor mass (Da) for each scan list. Ions are identified that differ in *m/z* by the expected Δ, assuming the fragment ions are either singly or doubly charged. As a further level of confidence, the mass-shifted ions that can be predicted from the exact-matched ions are highlighted on the plot. The expected PTM-containing fragment ion is equal to the difference between the precursor mass and an exact-matched ion. The ion in the heavy-labelled peptide can be calculated from the light peptide, by accounting for the mass of the isotopic label:

$$\begin{array}{c} m_{H}^{c}=m_{L}^{p}-m^{M}+△\pm T\\ m_{H}^{c}=\left[\frac{m_{L}^{p}}{z_{L}^{p}}z_{L}^{p}-z_{L}^{p}H^{+}\right]-\left[\frac{m^{M}}{z^{M}}z^{M}-z^{M}H^{+}\right]+\Delta \pm T \end{array}$$

where the mass of the heavy complementary ion, *(*$m_{H}^{c}$*)*, is the mass of the light precursor ($m_{L}^{p}$), plus the difference between the light and heavy label (Δ), less the mass of an exact-matched ion ($m^{M}$). As the charge (*z*), on a fragment ion is not always known, we assume it is either +1 or +2 and calculate both predicted ions accordingly. The predicted ions are matched within a tolerance (*T*) (0.8 Da by default). All matched peak lists are exportable as csv’s.

Peak labels are customizable; to prevent over-crowding, by default, the maximum number of labels plotted is 15 and are at least 15 Da apart. Plots can be saved as either a portable network graphic (png) or JPEG (jpg) with the output size set at 19.4x15cm by default.

## 3 Evaluation

Within our group, we use this program as a rapid visual aid for comparing two MS/MS spectra in the identification of protein methylation. Following culture with heavy-labelled methionine, methyl groups are present as a mixture of CH_3_ and ^13^CD_3_, thus co-eluting peptides are separated by a + 4Da shift. Often, we find that whilst MS/MS spectra are acquired for both heavy and light peptides, only 1 or neither may be assigned by database searching (Hart-Smith *et al.*, 2016). For these MS/MS pairs, we wish to visually ascertain if they are indeed the same methylated peptide or not. For example, Figure 1C and D shows the MS/MS of two doubly-charged peptides with a mass shift of 2 *m/z* (4 Da). Database searching only identified the heavy-labelled peptide (733 *m/z*) (Fig. 1B), despite MS/MS being present for both (Fig. 1C). Initial comparison of the MS/MS spectra is not conclusive—as the spectra for 731 *m/z* has more low abundance ions and more peaks around 1000 *m/z* compared to the spectra for 733 *m/z*. We then visualize the peaks that are the same between the two spectra, mass-shifted and predicted from the exact-matched ions (Fig. 1D).

We can see that exact-matched and mass-shift matches are present throughout the spectra and the relative intensities between them are similar. Furthermore, the higher-confidence matches—those predicted from the exact-mass matches—correspond to the fragment masses that contain the monomethyl-lysine in the annotated spectra. For example, the highlighted peak at *m/z* 964 or 968 in the light or heavy spectra respectively (Fig. 1D) is annotated as the y7 fragment (‘DFK_{me}_TDLR’) (Fig. 1B).

This visualization allows for easy identification of matched peaks and the identification of the mass-shifted ion series providing additional confidence to the annotated spectrum. Similarly, identifying the mass-shifted peaks in this manner provides information for *de novo* sequencing.

The peak list and optional inputs used to generate these figures are provided in Supplementary Material.

## 4 Conclusion

We have developed a simple to use tool for comparing two fragment ion peak lists for peptides with isotopically labelled PTMs. The application could also be used to evaluate heavy/light SILAC pairs, assess peptide assignment from database searching and aid *de novo* sequencing. The software is open source and free to use from https://harrywhitwell.shinyapps.io/hilight-ptm/

## Funding

This work was supported by CRUK (C33325/A23619).

*Conflict of Interest*: none declared.

## Supplementary Material

btz654_Supplementary_Data

## References

[btz654-B1] Chen X. et al (2015) Quantitative proteomics using SILAC: principles, applications, and developments. Proteomics, 15, 3175–3192. 26097186 10.1002/pmic.201500108

[btz654-B2] Cox J. et al (2011) Andromeda: a peptide search engine integrated into the MaxQuant environment. J. Proteome Res., 10, 1794–1805. 21254760 10.1021/pr101065j

[btz654-B3] Evertts A.G. et al (2013) Quantitative dynamics of the link between cellular metabolism and histone acetylation. J. Biol. Chem., 288, 12142–12151. 23482559 10.1074/jbc.M112.428318 PMC3636898

[btz654-B4] Hart-Smith G. et al (2016) Large scale mass spectrometry-based identifications of enzyme-mediated protein methylation are subject to high false discovery rates. Mol. Cell. Proteomics, 15, 989–1006. 26699799 10.1074/mcp.M115.055384 PMC4813715

[btz654-B5] Ong S.-E. et al (2004) Identifying and quantifying in vivo methylation sites by heavy methyl SILAC. Nat. Methods, 1, 119–126. 15782174 10.1038/nmeth715

[btz654-B6] Zee B.M. et al (2010) Global turnover of histone post-translational modifications and variants in human cells. Epigenet. Chromat., 3, 22. 10.1186/1756-8935-3-22 PMC3004898 21134274

