# Supplementary material for: HiLight-PTM: an online application to aid matching peptide pairs with isotopically labelled PTMs
Source: Bioinformatics. 2019 Aug 19;36(3):938–9. doi: 10.1093/bioinformatics/btz654 (PMC9883675; doi:10.1093/bioinformatics/btz654)
Supplement: btz654_Supplementary_Data [file bioinformatics_36_3_938_s2.zip › SupplementaryFigure.docx]

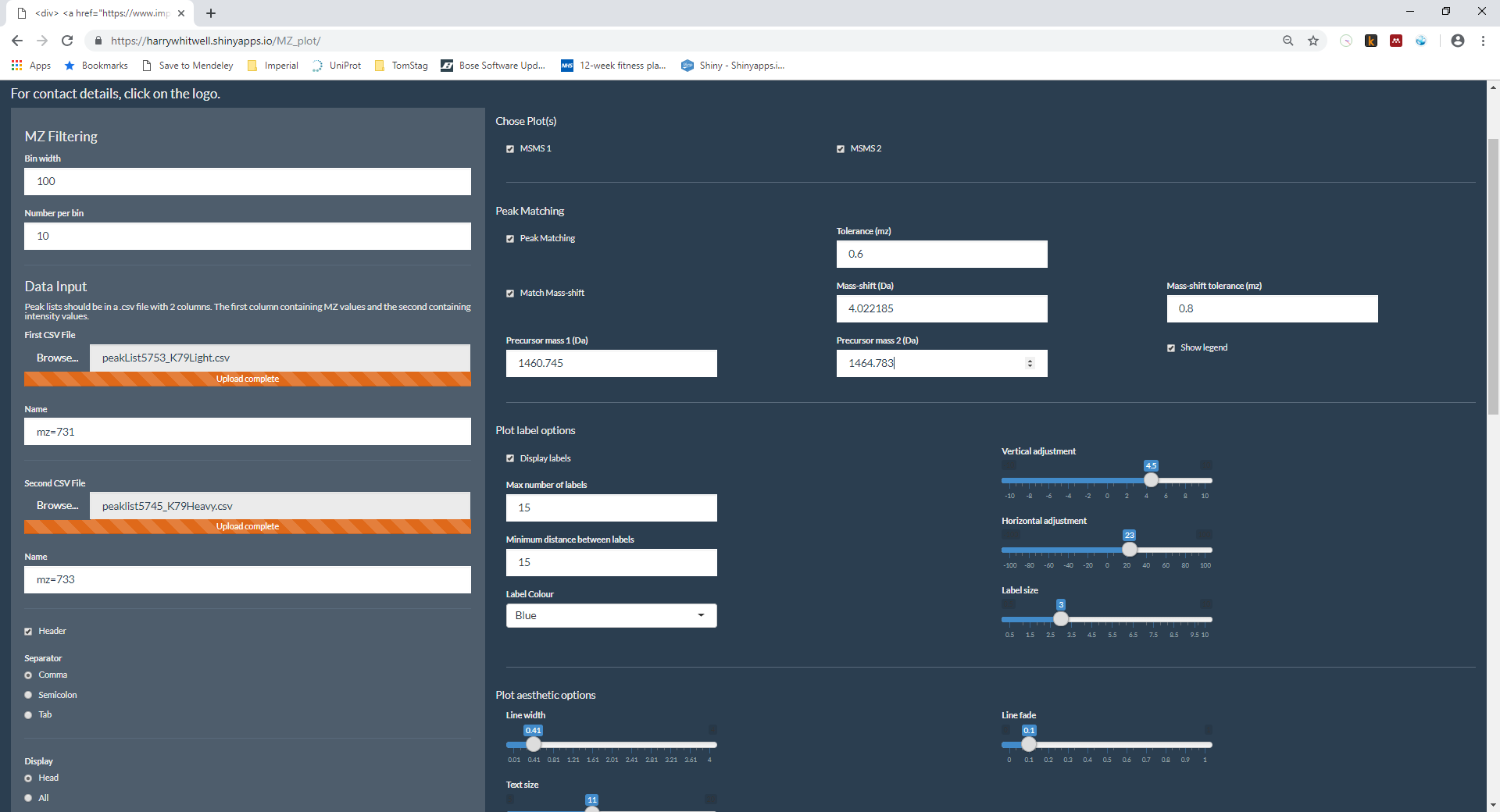


| MZ Filtering |  |
| --- | --- |
| Bin width | 100 |
| Number per bin | 10 |
| **Chose Plot** |  |
| MSMS 1 | Checked |
| MSMS 2 | Checked |
| **Peak Matching** |  |
| Peak Matching | Checked |
| Tolerance(mz) | 0.4 |
| Match Mass-shift | Checked |
| Mass-shift(Da) | 4.022185 |
| Mass-shift tolerance(mz) | 0.8 |
| Precursor mass 1 (Da) | 1460.745 |
| Precursor mass 2 (Da) | 1464.783 |
| Show legend | Unchecked |
| **Plot label options** |  |
| Display labels | Checked |
| Max number of labels | 15 |
| Minimum distance between labels | 15 |
| Label Colour | Blue |
| Vertical adjustment | 4.5 |
| Horizontal adjustment | 23 |
| Label size | 3 |
| **Plot aesthetic options** |  |
| Line width | 0.41 |
| Text size | 11 |
| Line fade | 0.1 |
| **Save plot options** |  |
| Plot width (cm) | 19.4 |
| Plot height (cm) | 15 |
